# The effect of depression, anxiety, and cognitive flexibility on pregnancy outcomes in women with unexplained infertility

**DOI:** 10.5935/1518-0557.20260032

**Published:** 2026

**Authors:** Kübra Dilşad Sönmez Özgür, Beyazıt Garip, Kazım Emre Karaşahin

**Affiliations:** 1 Department of Obstetrics and Gynecology, SBÜ Gülhane Faculty of Medicine, Ankara, Turkey; 2 Department of Psychiatry, SBÜ Gülhane Training and Research Hospital, Ankara, Turkey

**Keywords:** anxiety, cognitive flexibility, depression, unexplained infertility, Wisconsin Card Sorting Test, WCST

## Abstract

**Objective:**

This study aimed to investigate the interaction between depression, anxiety, and cognitive flexibility in women with unexplained infertility and to evaluate the impact of these psychological factors on pregnancy outcomes following assisted reproductive treatments (ART). The study also sought to determine whether cognitive flexibility could serve as a potential neuropsychological determinant influencing reproductive success.

**Methods:**

A total of 120 women aged 20-40 years were enrolled: 66 with unexplained infertility and 54 healthy controls with spontaneous conception. The infertility group underwent assisted reproductive treatments (ART) and was divided into pregnant (n=35) and nonpregnant (n=31) subgroups. Depression, anxiety, and cognitive flexibility were assessed using Beck Depression Inventory (BDI), Beck Anxiety Inventory (BAI), and the State-Trait Anxiety Inventory (STAI-I/II) and Wisconsin Card Sorting Test (WCST).

**Results:**

Depression scores significantly decreased in women who conceived after ART (*p*=0.012), whereas both depression and anxiety levels increased in those who did not achieve pregnancy (*p*<0.001). No significant changes were observed in the control group (*p*≥0.050). Women who conceived demonstrated significantly higher WCST category completion scores compared to those who did not conceive (*p*=0.046), while no group difference was found in perseverative error counts.

**Conclusion:**

Cognitive flexibility may influence treatment success in unexplained infertility. Higher WCST category completion in women who conceived suggests that supporting psychological resilience and adaptive coping may contribute to improved emotional well-being and reproductive outcomes.

## INTRODUCTiON

Infertility is defined as the inability to achieve pregnancy after at least one year of regular, unprotected sexual intercourse (Zegers-Hochschild *et al.*, 2017). Detectable causes can be identified in approximately 85% of infertile couples, most commonly related to ovulatory dysfunction, male factors, or tubal pathology (Carson & Kallen, 2021). Unexplained infertility refers to cases in which no anatomical, hormonal, or genetic abnormalities are detected despite standard evaluations, representing about 10-30% of all infertility cases (Datta *et al.*, 2016; Stewart *et al.*, 2019). Infertility, a prevalent clinical condition, affects individuals both physiologically and psychologically, and its incidence has increased in recent decades due to lifestyle changes, delayed childbearing, and socioeconomic factors (Datta *et al.*, 2016; Stewart *et al.*, 2019).

Psychological distress is highly common among infertile women. Several studies have reported elevated levels of anxiety, stress, and depression compared with the general population (Monga *et al.*, 2004). Common symptoms include irritability, reduced self-esteem, and emotional instability, which may further impair treatment adherence and overall life satisfaction. Previous evidence indicates that these psychological factors can negatively affect reproductive outcomes and decrease pregnancy rates (Monga *et al.*, 2004; Kamışlı *et al.*, 2021; Kargın & Ünal, 2011; Szkodziak *et al.*, 2020).

Cognitive flexibility refers to the mental ability to adapt thoughts and behaviors in response to changing internal or external demands (Joseph & Whirledge, 2017; Gunuc & Koylu, 2023). This capacity enables individuals to modify coping strategies, regulate emotions, and maintain adaptive responses under stress. Impaired cognitive flexibility and executive dysfunction are frequently associated with depressive and anxiety disorders, which are also prevalent among infertile women (Joseph & Whirledge, 2017; Gunuc & Koylu, 2023). Because cognitive flexibility supports emotional regulation and adaptive coping, it may play a crucial role in how individuals manage infertility-related stress and influence their reproductive success. Cognitive flexibility may support emotional regulation and adaptive coping, facilitating the management of infertility-related psychological stress and potentially improving treatment adherence. Despite growing recognition of the psychological burden of infertility, the neurocognitive mechanisms underlying these associations remain poorly understood. In particular, no prior study has examined the relationship between cognitive flexibility objectively measured using the WCST and pregnancy outcomes among women with unexplained infertility.

The role of cognitive flexibility in reproductive success offers a new perspective for understanding the psychological dimensions of infertility at a neuropsychological level.

Therefore, the interactions among depression, anxiety, and cognitive flexibility were evaluated in women with unexplained infertility, and the effects of these psychological factors on pregnancy outcomes following assisted reproductive treatments were investigated. According to the current literature, this is among the first studies to objectively address cognitive flexibility as a potential neuropsychological determinant influencing reproductive success in this population. We hypothesized that higher cognitive flexibility, together with lower levels of depression and anxiety, would be associated with more favorable reproductive outcomes in women undergoing ART.

Despite extensive evidence highlighting the psychological burden of infertility, no previous research has simultaneously examined the combined influence of depression, anxiety, and objectively measured cognitive flexibility on reproductive outcomes in women with unexplained infertility. Therefore, the present study was designed to address this knowledge gap by evaluating how these psychological factors relate to pregnancy achievement after ART. Enhanced cognitive flexibility and improved stress-related emotional regulation may be associated with more favorable reproductive outcomes. Clarifying these associations may help identify cognitive flexibility as a potential psychological target to support treatment success in infertility care.

## MATERiALS AND METHODS

**Figure 1:**
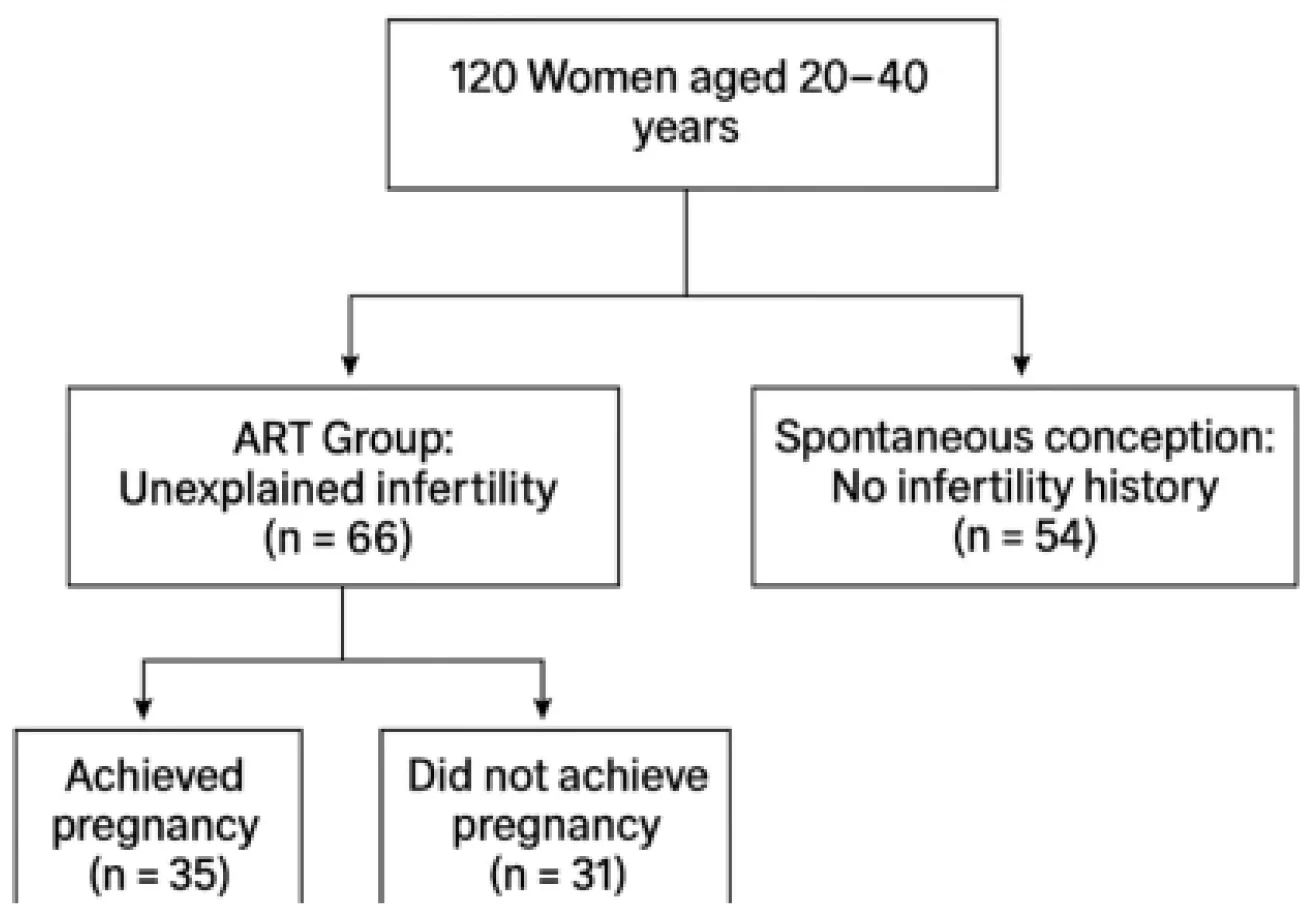
The flow diagram of the study

This prospective case controlled study was conducted between June 2023 and February 2024 at the infertility outpatient clinic and included 120 women aged 20-40 years. Participants were divided into two main groups: women diagnosed with unexplained infertility who underwent ART (n=66) and women who conceived spontaneously without any infertility history (n=54). The patient group was further subdivided according to treatment outcomes into those who achieved pregnancy and those who did not (Figure 1). Pregnancy was confirmed by the presence of a gestational sac with fetal heartbeat on transvaginal ultrasonography performed 5-6 weeks after embryo transfer. Eligible participants were women aged 20-40 years with regular ovulatory cycles, normal hormonal profiles, and no structural abnormalities of the reproductive organs. Women with a history of psychiatric illness, chronic systemic or endocrine disease, use of antidepressant or antipsychotic medication, or male-factor infertility were excluded. The participants were informed about the nature and the possible outcomes of the study. Verbal consent and signed informed consent forms were obtained from the participants. Psychiatric evaluation was performed according to the Structured.Clinical Interview for DSM-5 Disorders (SCID-5-TR)

Controlled ovarian stimulation and treatment protocols were individualized according to patient characteristics, including age, ovarian reserve, body mass index, antral follicle count, and previous treatment response; therefore, a uniform stimulation regimen was not applied to all participants. Stimulation was initiated with recombinant follicle-stimulating hormone (rFSH) or human menopausal gonadotropin (hMG), and depending on the ovarian response, either a gonadotropin-releasing hormone (GnRH) antagonist or a modified short protocol was used. Oocyte maturation was triggered with human chorionic gonadotropin (hCG) or a GnRH agonist followed by oocyte retrieval 34-36 hours later, and embryo transfer was performed in accordance with institutional ART standards. Because treatment was personalized, the detailed dosage and duration of medications were not recorded in the study database. All participants completed the Beck Depression Inventory (BDI), Beck Anxiety Inventory (BAI), and the State-Trait Anxiety Inventory (STAI-I/II). All psychological assessments, including the WCST, were administered by the same psychiatrist in a standardized quiet environment (B.G).

WCST was used to objectively assess cognitive flexibility, set-shifting, and problem-solving ability. The computerized version validated previously (Çelik *et al.*, 2021). Was administered. During the task, participants matched stimulus cards to one of four key cards according to changing rules based on colour, shape, or number. The software automatically generated the WCST outcomes, including total correct responses, total errors, categories completed, perseverative errors, and conceptual level responses.

The minimum required sample size was calculated using G*Power 3.1 software, assuming an effect size of 0.5, α=0.05, and a power of 0.80, which indicated that at least 50 participants per group were necessary. A total of 66 women in the ART group and 54 in the control group were included. Since both main groups consisted of more than 50 participants, the required statistical power was achieved (power=0.780). The study protocol was approved by the Institutional Ethics Committee (Approval No: 2023/99, May 17, 2023), and written informed consent was obtained from all participants in accordance with the Declaration of Helsinki. Statistical analyses were performed using IBM SPSS Statistics version 25.0. The Shapiro-Wilk test was used to assess normality. Continuous variables were expressed as mean±standard deviation or median [min-max], and group comparisons were performed using the independent-samples t-test or Mann-Whitney U test, as appropriate. Categorical variables were analyzed using the chi-square test. Bonferroni correction was applied for multiple comparisons, and *p*<0.050 was considered statistically significant. Effect sizes (Cohen’s d for t-tests and η^2^ for ANOVA) were calculated to quantify the magnitude of group differences.

## RESULTS

A total of 120 women were included in the study: 66 in the assisted reproductive treatment (ART) group and 54 in the control group. The ART group was further divided into women who achieved pregnancy (n=35) and those who did not (n=31). The sample size was sufficient to ensure adequate statistical power. No significant differences were found between the groups in age, body mass index (BMI), or education level, confirming comparable baseline characteristics.

In women who achieved pregnancy after ART, depression scores significantly decreased from baseline to week 12 (*p*=0.012), corresponding to an approximately 41% reduction in depressive symptoms. Anxiety and state-trait anxiety scores also showed a mild but non-significant decreasing trend (each *p*>0.050). No similar change was observed in the control group (*p*>0.05) (Table 1).

**Table 1:** Comparison of baseline, 6th week, and 12th week scale scores in the ART group

ART	Baseline	Week 6 ^6^	Week 12 ^12^	*p*	Post hoc test
	Mean±SD Median (Min-Max)	Mean±SD Median (Min-Max)	Mean±SD Median (Min-Max)		
Depression	15.54±12.58 13 (0-45)	9.49±8.16 8 (0-34)	9.20±7.63 7 (0-34)	**0.005 ^d^**	0-6 *p*=0.069 0-12 *p*=0.010 6-12 *p*=1.000
Anxiety	12.91±11.64 9 (0-42)	11.97±11.63 9 (0-62)	10.00±10.90 7 (0-62)	0.108 ^d^	
STAI-1	42.29±7.21 41 (26-61)	44.49±7.02 43 (34-62)	42.51±6.90 41 (32-61)	0.063 ^f^	
STAI 2	45.57±5.35 45 (35-56)	45.20±6.24 45 (30-61)	43.97±6.54 42 (30-59)	0.345 ^f^	

Data are expressed as Mean±SD or Median [Min-Max].

p<0.05 was considered statistically significant.

ART: Assisted Reproductive Treatment ; STAI: State-Trait Anxiety Inventory.

Post hoc comparisons represent pairwise differences between time points (0-6 weeks, 0-12 weeks, 6-12 weeks).

Conversely, in women who did not achieve pregnancy after ART, both depression and anxiety scores significantly increased following treatment (each *p*<0.001), while no significant change was observed in state-trait anxiety levels (*p*>0.05). These findings indicate that unsuccessful treatment was associated with worsening emotional distress and reduced psychological well-being, as reflected by higher post-treatme nt Beck Depression Inventory (BDI) and Beck Anxiety Inventory (BAI) scores (Table 2).

**Table 2:** Comparison of baseline and post-treatment scale scores in patients with unexplained infertility

Unexplained Infertility^b^ (n=31)	Baseline	Post-Treatment	*p*
	Mean±SD Median (Min-Max)	Mean±SD Median (Min-Max)	
Depression	7.77±5.07 7 (3-27)	11.77±8.30 10 (3-33)	**<0.001 ^c^**
Anxiety	7.52±6.41 5 (1-25)	10.45±7.00 8 (3-29)	**<0.001 ^c^**
STAI-1	43.74±6.20 43 (32-57)	43.39±5.91 44 (33-58)	0.767 ^h^
STAI-2	48.13±5.74 48 (32-62)	48.77±4.93 48 (41-58)	0.562 ^h^

Data are expressed as Mean±SD.

p<0.050 considered statistically significant.

STAI: State-Trait Anxiety Inventory.

According to the WCST results, no significant differences were found among the groups in total correct responses, total errors, or perseverative errors (*p*>0.050). However, within the ART subgroup, women who achieved pregnancy completed a significantly higher number of categories compared with those who did not (*p*=0.046). This finding indicates that enhanced cognitive flexibility may contribute to better adaptive functioning and improved treatment outcomes. Considering that cognitive flexibility facilitates emotional regulation and problem-solving under stress, these results provide a novel perspective linking neuropsychological adaptability with reproductive success (Table 3).

**Table 3:** Comparison of WCST scores among patients in the ART, unexplained infertility, and control groups.

	ARTª (n=35)	Unexplained Infertility^b^ (n=31)	Control ͨ (n=64)	*p*	Post hoc test
	Mean±SD Median (Min-Max)	Mean±SD Median (Min-Max)	Mean±SD Median (Min-Max)		
WCST Total Response	94.77±23.26 92 (18-128)	101.16±20.23 98 (70-128)	106.83±21.91 110 (70-128)	0.057 ^b^	
WCST Total Error	20.77±12.18 18 (7-59)	29.61±21.98 20 (5-80)	32.43±22.46 28 (5-88)	0.066 ^b^	
WCST Category Count	5.97±2.96 6 (2-22)	5.39±1.11 6 (2-6)	4.94±1.43 6 (0-6)	**0.046 ^b^**	a-b *p*=1.000 a-c *p*=0.020 b-c *p*=0.284
WCST Perseverative Error	12.09±5.56 11 (4-27)	16.13±11.73 12 (4-47)	16.93±15.30 11 (4-97)	0.531 ^b^	

Data are expressed as Mean±SD or Median [Min-Max].

p<0.050 considered statistically significant.

WCST: Wisconsin Card Sorting Test; ART: Assisted Reproductive Treatment.

a: comparison between ART and unexplained infertility;

b: comparison between ART and control;

c: comparison between unexplained infertility and control.

Overall, depressive symptoms decreased in the ART-pregnancy subgroup and increased in the ART-nonpregnancy subgroup. WCST performance showed a significant difference between subgroups: the number of completed categories was higher among women who achieved pregnancy.

## DiSCUSSiON

Infertility is frequently accompanied by elevated levels of depression, anxiety, hopelessness, and a marked decline in quality of life (Monga *et al.*, 2004; Kamışlı *et al.*, 2021; Kargın & Ünal, 2011). This psychological burden may impair stress regulation, emotional adjustment, and treatment adherence, thereby indirectly undermining reproductive success (Szkodziak *et al.*, 2020). Cognitive flexibility, a core component of executive functioning, plays a central role in helping individuals adapt to uncertainty, manage emotional distress, and maintain effective coping strategies (Joseph & Whirledge, 2017; Gunuc & Koylu, 2023). Therefore, integrating psychological well-being and cognitive processes into infertility management has substantial clinical relevance.

Psychological stress associated with infertility has been shown to dysregulate the hypothalamic-pituitary-adrenal (HPA) axis, leading to alterations in neuroendocrine functioning and ovulatory disturbances (Oftedal *et al.*, 2023; Hohl & Dolcos, 2024). These mechanisms may partly explain how emotional distress influences reproductive outcomes and underscore the importance of incorporating stress-management strategies into infertility care.

Previous research has reported higher levels of anxiety and depression among women undergoing ART compared with those who conceive spontaneously (DelRosso *et al.*, 2022). Consistent with these findings, the present study demonstrated a significant reduction in post-treatment depression scores among women who conceived after ART. This improvement may reflect the emotional relief and psychological adjustment that often accompany successful conception, supporting the role of psychosocial support and resilience-oriented interventions during infertility treatment. To the best of our knowledge, no previous study has simultaneously examined depression, anxiety, and objectively measured cognitive flexibility as predictors of pregnancy outcomes among women with unexplained infertility, highlighting the novelty and clinical relevance of the present research.

According to the WCST results, women who achieved pregnancy exhibited significantly higher category completion scores than those who did not, whereas no significant difference was observed in perseverative error counts. These findings suggest that category completion may represent a more sensitive measure of cognitive flexibility than perseverative errors alone, aligning with previous studies linking cognitive flexibility to executive functioning and adaptive problem solving abilities (Li, 2004; Lehto, 1996).

The current findings also support broader evidence indicating that cognitive flexibility may function as an important psychological resource for adapting to infertility-related stressors (Orakcı, 2021; Barceló *et al.*, 1997). Individuals with higher cognitive flexibility tend to demonstrate better emotional regulation and more effective problem solving under stressful conditions, which may facilitate healthier coping during infertility treatment. Incorporating assessments of executive functioning into infertility care may therefore provide clinicians with valuable insight into patients’ psychological strengths and challenges, supporting more individualized and comprehensive treatment planning.

Although perseverative errors are frequently used as an indicator of WCST performance, they may not fully capture the broader construct of cognitive adaptability. The difference in category completion between subgroups observed in this study suggests that this WCST component may better reflect the flexibility-related aspects of executive functioning in women undergoing infertility treatment (Barbieri, 2019). These findings highlight the importance of adopting a multidimensional approach when evaluating executive functions in this population.

Several limitations should be acknowledged. The single-center design and limited sample size may restrict the generalizability of the findings. Psychological variables were assessed using self-report measures, which may introduce response bias. Additionally, the WCST was administered at a single time point, preventing the evaluation of changes in cognitive performance over time. Hormonal profiles and biochemical stress markers were not measured; therefore, direct associations between HPA axis activity and cognitive parameters could not be established. Future multicenter, longitudinal studies incorporating neuroendocrine biomarkers and targeted psychological assessments are warranted to clarify these relationships.

This study suggests that cognitive flexibility may be a decisive psychological factor influencing treatment success in women with unexplained infertility. Among those undergoing assisted reproductive treatments, no significant difference was found in perseverative error counts; however, women who conceived demonstrated significantly higher category completion scores on the WCST. These findings indicate that category completion may better reflect the cognitive mechanisms underlying treatment responsiveness. Furthermore, higher cognitive flexibility together with lower depression levels may enhance emotional resilience, coping capacity, and treatment adherence during infertility management (Baddeley, 1999). Strengthening cognitive flexibility, alongside psychosocial coping mechanisms, may further improve both psychological well-being and clinical outcomes. These results highlight the importance of addressing not only physiological but also cognitive and psychological factors within a holistic infertility care framework. Incorporating the assessment and support of cognitive flexibility into routine infertility protocols may therefore contribute to improved emotional well-being and treatment responsiveness.

## CONCLUSiON

This study suggests that cognitive flexibility may be a decisive psychological factor influencing treatment success in women with unexplained infertility. Among those undergoing assisted reproductive techniques, no significant difference was observed in perseverative error counts; however, women who conceived demonstrated markedly higher numbers of completed categories on the WCST. Higher cognitive flexibility together with lower levels of depression may enhance emotional resilience, coping capacity, and treatment adherence. Integrating psychosocial support and cognitive assessments into infertility care may contribute to improved emotional well-being and clinical outcomes. Larger multicenter longitudinal studies are warranted to further clarify the contribution of cognitive flexibility to reproductive outcomes. These findings provide a clinical rationale for incorporating cognitive flexibility focused psychological evaluation and support into routine infertility treatment protocols. Future multicenter studies with larger cohorts are warranted to further clarify the contribution of cognitive flexibility to psychological evaluation and support during infertility treatment protocols.

## References

[r1] Baddeley AD. (1999). East.

[r2] Barbieri RL., Strauss JF, Barbieri RL (2019). Yen and Jaffe’s Reproductive Endocrinology.

[r3] Barceló F, Sanz M, Molina V, Rubia FJ. (1997). The Wisconsin Card Sorting Test and the assessment of frontal
function: a validation study with event-related potentials. Neuropsychologia.

[r4] Carson SA, Kallen AN. (2021). Diagnosis and Management of Infertility: A Review. JAMA.

[r5] Çelik S, Oğuz M, Konur U, Köktürk F, Atasoy N. (2021). Comparison of computerized and manual versions of the Wisconsin
Card Sorting Test on schizophrenia and healthy samples. Psychol Assess.

[r6] Datta J, Palmer MJ, Tanton C, Gibson LJ, Jones KG, Macdowall W, Glasier A, Sonnenberg P, Field N, Mercer CH, Johnson AM, Wellings K. (2016). Prevalence of infertility and help seeking among 15 000 women and
men. Hum Reprod.

[r7] DelRosso LM, Vega-Flores G, Ferri R, Mogavero MP, Diamond A. (2022). Assessment of Executive and Cognitive Functions in Children with
Restless Sleep Disorder: A Pilot Study. Brain Sci.

[r8] Gunuc S, Koylu EO. (2023). Investigation of the Relationships Between Beck
Depression/Anxiety Scores and Neuropsychological Tests Scores with Lifestyle
Behaviors in the Context of Neuroplasticity and Neurogenesis
Approach. Neuroscience.

[r9] Hohl K, Dolcos S. (2024). Measuring cognitive flexibility: A brief review of
neuropsychological, self-report, and neuroscientific
approaches. Front Hum Neurosci.

[r10] Joseph DN, Whirledge S. (2017). Stress and the HPA Axis: Balancing Homeostasis and
Fertility. Int J Mol Sci.

[r11] Kamışlı S, Terzioğlu C, Bozdağ G. (2021). The psychological health of women with infertility: hopelessness,
anxiety and depression levels. J Psy Nurs.

[r12] Kargın M, Ünal S. (2011). İnfertil bireylerde umutsuzluğun
belirlenmesi. Yeni Symp.

[r13] Lehto J. (1996). Are executive function tests dependent on working memory
capacity?. Quart J Exp Psychol Sect A.

[r14] Li CSR. (2004). Do schizophrenia patients make more perseverative than
non-perseverative errors on the Wisconsin Card Sorting Test? A meta-analytic
study. Psychiatry Res.

[r15] Monga M, Alexandrescu B, Katz SE, Stein M, Ganiats T. (2004). Impact of infertility on quality of life, marital adjustment, and
sexual function. Urology.

[r16] Oftedal A, Tsotsi S, Kaasen A, Mayerhofer LJK, Røysamb E, Smajlagic D, Tanbo TG, Bekkhus M. (2023). Anxiety and depression in expectant parents: ART versus
spontaneous conception. Hum Reprod.

[r17] Orakcı Ş. (2021). Exploring the relationships between cognitive flexibility,
learner autonomy, and reflective thinking. Think Skills Creat.

[r18] Stewart JD, Pasternak MC, Pereira N, Rosenwaks Z. (2019). Contemporary Management of Unexplained
Infertility. Clin Obstet Gynecol.

[r19] Szkodziak F, Krzyżanowski J, Szkodziak P. (2020). Psychological aspects of infertility. A systematic
review. J Int Med Res.

[r20] Zegers-Hochschild F, Adamson GD, Dyer S, Racowsky C, de Mouzon J, Sokol R, Rienzi L, Sunde A, Schmidt L, Cooke ID, Simpson JL, van der Poel S. (2017). The International Glossary on Infertility and Fertility Care,
2017. Fertil Steril.

